# SwiftNINJA steerable microcatheter: a new kid on the block for selective catheterization of vascular and valvular congenital lesions

**DOI:** 10.3389/fcvm.2023.1322787

**Published:** 2023-12-04

**Authors:** Raymond N. Haddad, Ahmed Adel Hassan, Mahmoud Al Soufi, Mohamed Kasem

**Affiliations:** ^1^ M3C-Necker, Necker-Enfants Malades University Hospital, Assistance Publique – Hôpitaux de Paris (AP-HP), Paris, France; ^2^ Department of Pediatric Cardiology, Heart Centre of Excellence, Al Jalila Children’s Speciality Hospital, Dubai, United Arab Emirates

**Keywords:** acute angle branch, congenital heart disease, selective catheterization, steerable microcatheter, transcatheter interventions

## Abstract

**Background:**

SwiftNINJA (Merit Medical Systems, USA) is a novel steerable microcatheter intended for coronary and peripheral vascular interventions. We evaluate and report the first use of SwiftNINJA in pediatric catheterization of congenital heart defects (CHDs).

**Methods:**

We performed a retrospective clinical data review of children with CHDs in whom SwiftNINJA was used during cardiac catheterization between April 2022 and June 2023. Utility, application, and standard safety were described comprehensively.

**Results:**

We identified 19 patients (78.9% males) with a median age of 5.3 months (IQR, 2–13.9), and a median weight of 5.3 kg (IQR, 4–7.7). 36.8% of the catheterizations were transarterial and 78.9% were interventional. SwiftNINJA was applied upfront in 3/19 patients to cannulate precisely the right pulmonary artery and eliminate the risk of dislocating a freshly implanted left pulmonary flow restrictor. In 16/19 patients, SwiftNINJA was applied after a median of 5 (IQR, 5–7) failed catheterization attempts using various combinations of catheters, microcatheters, and wires to cannulate challenging vasculature in seven, engage the lumen of stented vessels in five, cross complex aortic valve stenosis in three, and cross an apical ventricular septal defect in one patient with Damus-Kaye-Stansel repair. After the SwiftNINJA application, catheterization was done from the first attempt in 12/16 patients and from the second attempt in 4/16 patients. The median applied tip angulation was 90 degrees (IQR, 85–95). All procedures were completed successfully. No device malfunction or adverse events occurred.

**Conclusions:**

SwiftNINJA is a valuable addition for selective catheterization of challenging vasculatures or valvular anatomies in children with CHDs.

## 1. Introduction

The growing operator experience and the continuous progress in catheter and guidewire technology have significantly increased the procedural success rate of cardiac catheterization procedures in patients with congenital heart defects (CHDs). Despite this, vessel tortuosity, sharply angulated origin of the side branch, jailed vessels, and the presence of protruding endovascular stents within the lumen represent a real challenge for the operator by making access to the target lesion tricky. Until recently, all selective catheterizations were performed using a combination of conventional guiding catheters, microcatheters, and guidewires (1, 2). Conventional catheters have a predetermined shape that cannot be modified once applied in-vivo. Studies evaluating the utility of steerable catheters and microcatheters in transcatheter interventions have shown a reduction in failure rate, procedure time, fluoroscopy time, and amount of contrast required (3–7). The SwiftNINJA® (Merit Medical Systems, Inc., USA) is a recent steerable microcatheter (SM) that allows operators to modify the angle and shape of its tip in real-time (8, 9). This device has been reported exclusively in interventional radiology procedures, promising to improve the success and efficiency of super-selective vessel cannulation (2–12). In 2022, we introduced this new device to our armamentarium and employed it upfront in tricky situations or as a bailout option for selective catheterization in children with challenging anatomies. In this study, we evaluate the application, utility, and safety of SwiftNINJA in the transcatheter management of CHDs.

## 2. Patients and methods

### 2.1. Study design

We performed a retrospective clinical data review of all children with CHDs in whom a SwiftNINJA SM was used during diagnostic or interventional catheterization procedures between April 2022 and June 2023. Standard safety and procedural data were collected and analyzed comprehensively. All cases were discussed and approved during multi-disciplinary team meetings before the intervention. Approval from the institutional review board was obtained. Signed informed consent was obtained from the patient's legal guardians.

### 2.2. Device

**Figure 1:**
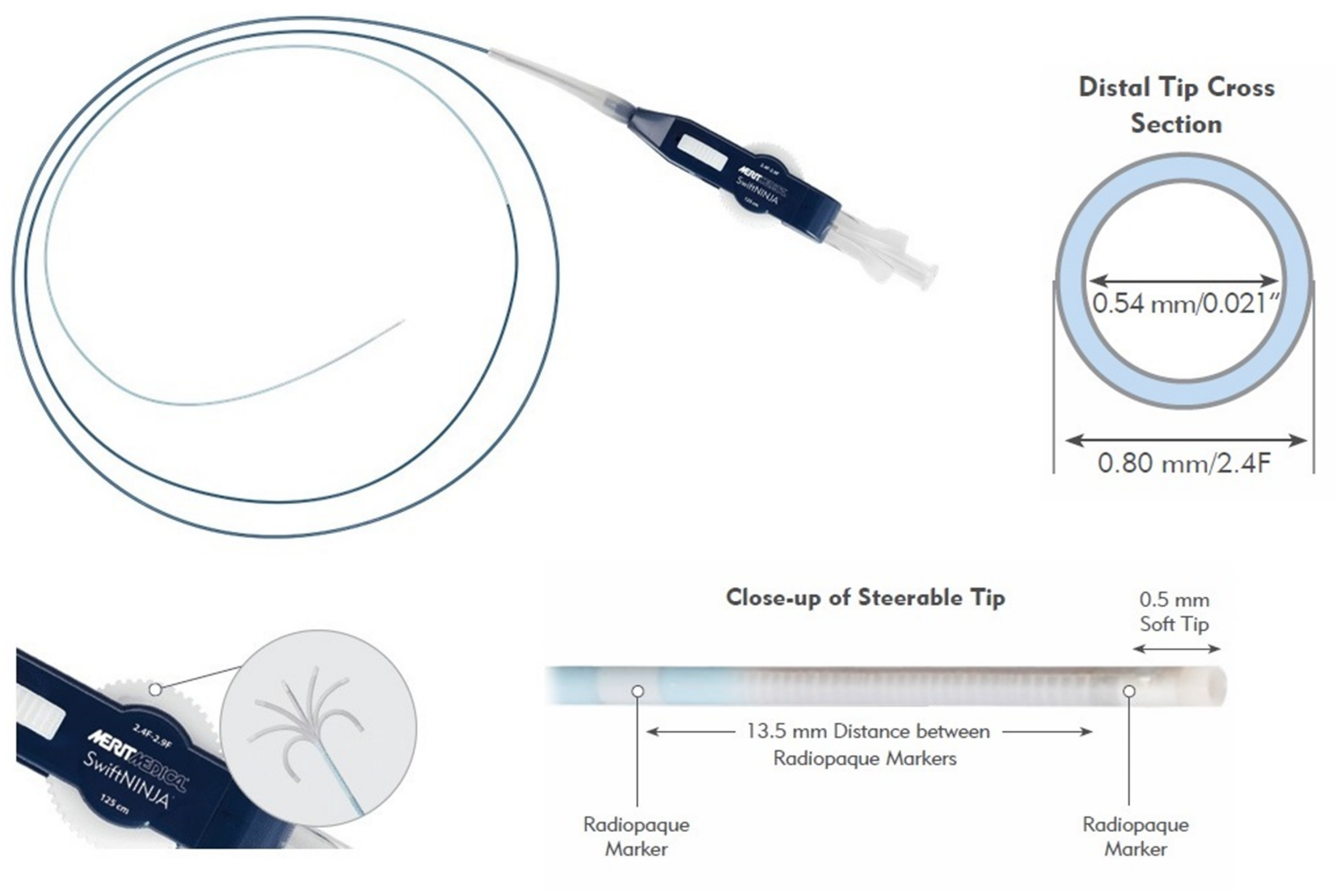
SwiftNINJA steerable nmicrocatheter.

SwiftNINJA® (Merit Medical Systems, Inc., USA) is a straight-tip SM designed with multi-dimensional steering control (Figure 1). SwiftNINJA has two operating wires running near the tip of the microcatheter to the steering dial in the hub. The articulation feature is activated by engaging the steering dial mechanism. This is done by pulling the white steering dial toward the luer connector until an audible clicking sound is heard. Turning the ergonomic steering dial will allow the distal tip of the catheter to articulate from a straight neutral position up to 180 degrees in opposing directions. Once the catheter is shaped into a curve, the catheter can be torqued and advanced through the vasculature. This provides “3D steering” as the catheter tip is moving in two planes simultaneously. Once shaped, the tip can be locked in the desired position by sliding the locking mechanism toward the luer connector. Once the target site is reached, the SwiftNINJA allows infusing diagnostic, embolic, or therapeutic material. There are two radiopaque marker bands for enhanced navigation and tip placement. The distance from the distal soft tip to the first marker band is 0.5 mm. The distance between the two marker bands is 13.5 mm. SwiftNINJA has a Tungsten-braided shaft and a hydrophilic coating on the distal 80 cm to enhance shaft support and provide pushability. The usable length of the catheter is 125 cm. The outer diameter is 2.9-Fr (0.97 mm) at the proximal portion and decreases to 2.4-Fr (0.80 mm) at the distal portion. The recommended inner diameter of the guiding catheter is 0.042–0.043 Inch (1.07–1.09 mm). The inner diameter of the catheter is 0.021 Inch (0.54 mm), taking a maximum guidewire of 0.018 Inch (0.46 mm). The maximum injection pressure is 1,000 psi (6,900 kPa). The maximum particle size deliverable through the SM is 700 µm and the maximum coil size is 0.018 inch.

### 2.3. Procedure

All procedures were performed under general anesthesia, intravenous heparinization, and biplane fluoroscopy guidance. Antibiotic prophylaxis was given when indicated.

We employed the SwiftNINJA upfront after baseline non-selective angiography to cannulate accurately, in one single attempt, a tricky right pulmonary artery and subsequently implant a microvascular plug-based bespoke pulmonary flow restrictor without touching the freshly implanted pulmonary flow restrictor in the left pulmonary artery (13). This eliminates the risk of mobilizing the left pulmonary flow restrictor in case the left pulmonary artery is cannulated unintentionally using standard pre-shaped hardware.

We also employed the SwiftNINJA as a bailout option when two senior operators spent at least 15 min of fluoroscopy (i.e., minimum of 5 attempts of 3 min of fluoroscopy each) trying to (1) cannulate complex vasculature (small vessels arising with an acute angle or at an angle close to 90° from the feeding vessel and tortuous vessel with at least two loops), (2) engage a challenging side branch jailed by a stent, (3) cannulate the lumen of stented vessels without crossing the struts of the protruding endovascular stent within the catheterization vascular course, (4) navigate the heart purposely through unusual routes, or (5) cross challenging intra-cardiac defects or stenotic aortic valves using various combinations of conventional pre-shaped catheters, microcatheters, and micro-wires.

When using a guidewire to advance the SwiftNINJA into the vasculature, the wire was pulled so it was proximal to the most proximal marker band before articulating the tip. While watching under fluoroscopy, the distal tip of the SwiftNINJA was deflected to the same angle of the vessel branch to help direct and advance the coronary wire and SwiftNINJA through the vasculature to the target lesion. Once the desired curve was achieved and the catheter tip was locked, the guidewire was advanced out the catheter tip into the vessel. The catheter tip was unlocked before advancing the catheter. Following successful vessel negotiation, the coronary wire was kept in place, the SwiftNINJA was returned to its straight configuration and removed. The coronary wire was used as a tutor for subsequent interventions or to advance a 4-Fr multipurpose Glidecath hydrophilic coated catheter (Terumo, Japan) to perform contrast injections or pressure measurements.

### 2.4. Follow-up

The patient's follow-up was performed according to the institutional protocol (clinical examination and transthoracic ultrasound). General and vascular access complications were looked at.

### 2.5. Statistical analyses

Statistical analyses were performed using SPSS, Version 22.0 for Macintosh (IBM, Armonk, NY, USA). Categorical variables were reported as frequency and percentage and continuous variables were represented as median with interquartile range.

## 3. Results

### 3.1. Baseline patients characteristics

We identified 19 patients (78.9% males) with a median age of 5.3 months (IQR, 2–13.9), and a median weight of 5.3 kg (IQR, 4–7.7). The patients' characteristics are outlined in Table 1.

**Table 1:** Patients clinical and procedural data.

CN	Gender	A/W/H	Disease/Clinical presentation	Cardiac catheterization procedure	Vascular Access	Attempted Catheters & wires	NOAb^a^	TA	NOAa*	FT
1	Male	11.5/7.6/70	Abernathy malformation Type II - Portal veins hypoplasia/Hyperbilirubinemia	Diagnostic	RFV	JR 3.5 - Straight CW	7	90	1	31
2	Female	9.8/6.5/67	Transposition of the great arteries - PA - RPA Narrowing – s/p DS/Cyanosis	Diagnostic (Pre-stage II surgery)	RFV	JR 3.5 - JR 1.5 - Glidecath MP - Progreat MC - Straight and J-tip CW	12	90	1	49
3	Female	2.4/2.2/45	Large 2 VSDs - ASD – arterial duct Pulmonary overflow - Heart failure	Bilateral PFR Implantation	RFV	Upfront use of SwiftNINJA®	–	130	1	22
4	Male	0.03/2.7/48	Critical AVS - borderline left ventricle - Parachute mitral valve - Restrictive atrial shunt	AVB	RAA	JR 3.5 - Glidecath MP - Progreat MC - Straight and J-tip CW	7	75	1	21
5	Male	17.6/9/79	PA-VSD - MAPCA - s/p BTS - s/p pulmonary outflow conduit (Unifocalisation)/Cyanosis	Pulmonary artery ballooning	RFV	JR 3.5 - Glidecath MP - Progreat MC - Straight and J-tip CW	6	75	1	75
6	Male	0.33/4.4/59	Severe AVS	AVB	RFA	JR 3.5 – MPA II - Straight CW	10	90	1	33
7	Male	31.93/10/83	Unbalanced AVSD - PA - s/p DS - s/p BTS/Cyanosis - RPA stenosis - DS stenosis	RPA ballooning + DS ballooning	RFV	JR 3.5 - JR 1.5 - Straight and J-tip CW	8	135	1	69
8	Male	1.7/7.2/58	Severe AVS	AVB	RAA	JR 3.5 - Straight CW	5	60	2	21
9	Male	7.7/7.9/70	DORV – PA - s/p BTS/Cyanosis	Diagnostic (Pre-stage II surgery)	RFA	JR 3.5 - Straight CW	6	135	1	19
10	Male	16.3/3.7/49	Left Atrial Isomerism - TOF - Abernathy malformation Type II/Cyanosis – Liver Failure	Abernathy Veins Partial occlusion – RVOT stenting	RFV	JR 3.5 - Glidecath MP and Cobra 2 - Straight CW	11	90	1	82
11	Male	2.5/4.3/57	PA - s/p DS/Cyanosis	DS ballooning	RFA	JR 3.5 - Glidecath MP - Straight CW	5	140	1	86
12	Male	0.2/3/50	Hypoplastic left heart/Heart Failure	Bilateral PFR Implantation	RFV	Upfront use of SwiftNINJA®	–	90	1	23
13	Male	23.3/10/81	TOF - s/p BTS - s/p TOF repair - s/p RPA stenting/RPA and LPA stenosis	RPA stent + LPA ballooning	RFV	JR 3.5 - Glidecath MP – Wedge – J-tip Terumo Wire 0.035-Inch - Straight CW	5	60	1	40
14	Male	29/5/69	PA - Tricuspid atresia - NRGA - s/p DS/Cyanosis - RPA narrowing	Assessment pre-stage II surgery + RPA ballooning	RFA	JR 3.5 - Glidecath MP - Straight CW	5	90	1	63
15	Female	0.5/3/42	Unbalanced AVSD - Hypoplastic aortic arch/Pulmonary overflow - Heart failure	Bilateral PFR Implantation	RFV	Upfront use of SwiftNINJA®	–	90	1	43
16	Male	5.3/5.3/49	PA-VSD - s/p DS/Cyanosis	Diagnostic (Pre-stage II surgery)	RFV	JR 3.5 - Glidecath MP - Straight CW	5	90	1	44
17	Male	25.2/9.5/74	Taussig Bing Anomaly - s/p DKS + aortic arch repair - Residual Apical VSD	Apical VSD closure	RFA	Cut Pigtail - JR 3.5 - Glidecath MP - Straight and J-tip CW	5	100	2	79
18	Male	6.3/4.4/56	Supra-cardiac TAPVD – s/p surgical repair/PV re-stenosis - Small ASD	PV Ballooning	RFV	JR 3.5 - Glidecath MP - Straight CW	5	90	2	47
19	Female	3.9/5.3/54	Intra-cardiac TAPVD – s/p surgical repair/PV re-stenosis - Small ASD	PV Ballooning	RFV	JR 3.5 - Glidecath MP - Straight CW	5	80	2	74

ASD, atrial septal defect; A/W/H, age (months)/weight (kg)/height (cm); AVB, aortic valve ballooning; AVS, aortic valve stenosis; AVSD, atrioventricular septal defect; BTS, blalock-taussig shunt; CN, case number; CW, coronary wire; DS, ductal stenting; DORV, double outlet right ventricle; FT, fluoroscopy time (min); FU, follow-up; JR, judkins right; LAA, left axillary artery; LPA, left pulmonary artery; MC, microcatheter; MP, multipurpose; NOAa, number of attempts (after SwiftNINJA application); NOAb, number of attempts (before SwiftNINJA application); NRGA, normally related great arteries; PA, pulmonary atresia; PV, pulmonary vein; RAA, right axillary artery; RFA, right femoral artery; RFV, right femoral vein; RIJV, right internal jugular vein; RPA, right pulmonary artery; RV, right ventricle; PFR, pulmonary flow restrictor; TA, tip angulation (degrees); TAPVD, total anomalous pulmonary venous drainage; TOF, tetralogy of fallot; VSD, ventricular septal defect.

^a^ One catheterization attempt was defined as 3 min of fluoroscopy.

### 3.2. Procedure

**Figure 2:**
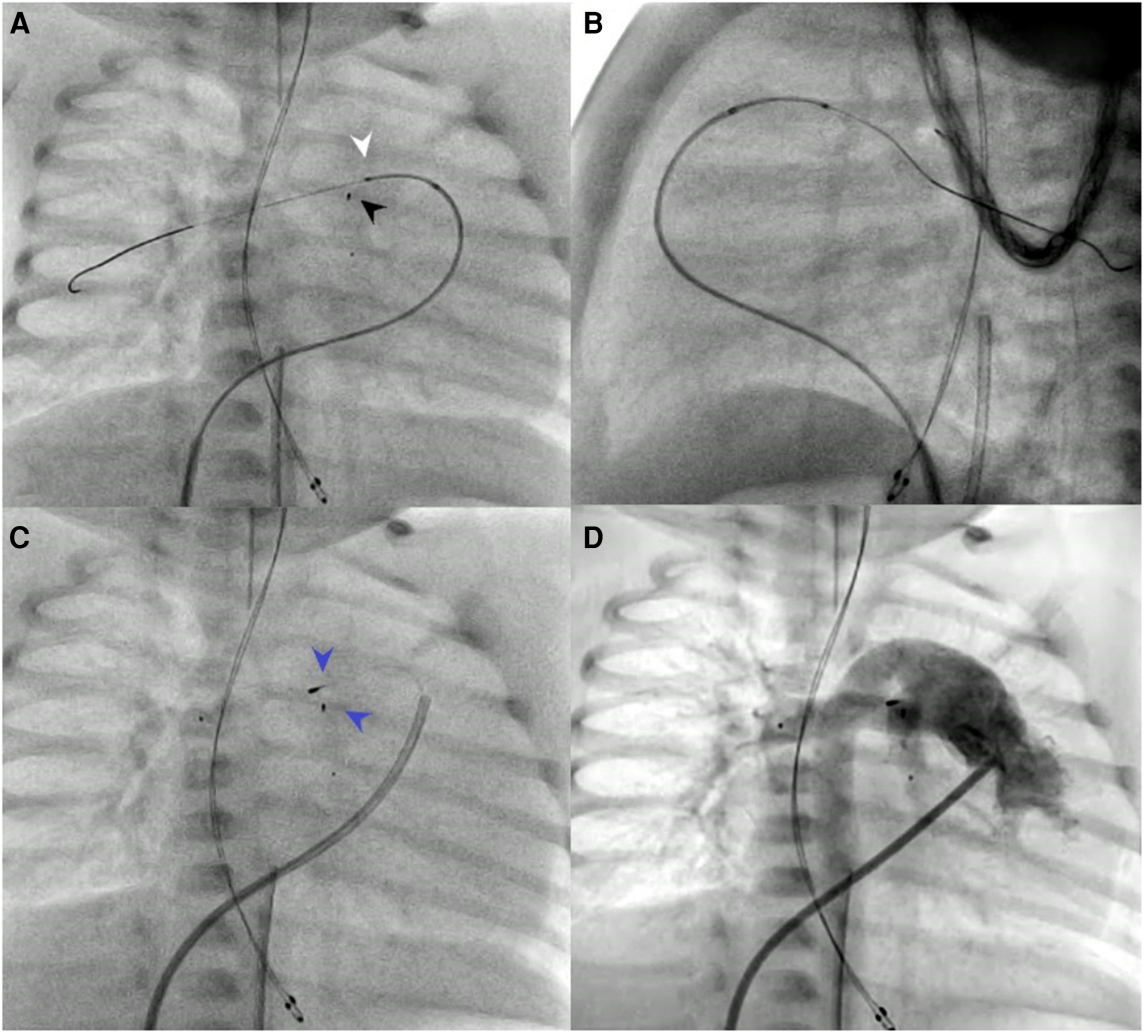
Patient no. 13. SwiftNINJA is articulated precisely to cannulate the origin of the right pulmonary artery opening (white pointed arrow) to make sure that the wire will not touch the proximal part of the freshly implanted left pulmonary flow restrictor (black pointed arrow) (**A,B**). Precise implantation of bilateral pulmonary flow restrictors (Blue pointed arrows) (**C,D**).

The cardiac catheterizations were transarterial in 36.8% of patients and interventional in 78.9% of patients. In three patients (no. 3, no. 12, and no. 15), the SwiftNINJA was applied upfront for precise cannulation of the right pulmonary artery and subsequent pulmonary flow restrictor implantation (Figure 2). In the other 16 patients, SwiftNINJA was applied after a median of 5 (IQR, 5–7) failed catheterization attempts using various combinations of catheters, microcatheters, and wires to rapidly cannulate challenging vasculature in seven, engage the lumen of stented vessels in five, cross complex aortic valve stenosis in three, and cross an apical ventricular septal defect in one.

In those 16 patients, the utilized pre-shaped 4-Fr diagnostic catheters included the Judkins right 3.5 coronary diagnostic catheters (Cordis, USA), Performa® pediatric Judkins right 1.5 catheters, Performa® multipurpose A1 catheter (Merit Medical Systems, USA), Cut Pigtail diagnostic catheter (Cordis, USA), and the Multipurpose or Cobra 2 Glidecath hydrophilic coated catheter (Terumo, Japan). The utilized conventional microcatheter was the 2.7-Fr Progreat® microcatheter (Terumo, Japan). The utilized wires included straight or J-tip 0.032 or 0.035-inch Radifocus® Hydrophilic Guidewire M (Terumo, Japan), Hi-Torque Balance Middle Weight (BMW) universal II 0.014-inch coronary wire (Abbott, USA), and SION blue 0.014 inch coronary wire (Asahi Intecc, Japan). After the SwiftNINJA application, catheterization was completed successfully from the first attempt in 12/16 patients and from the second attempt in 4/16 patients.

**Figure 3:**
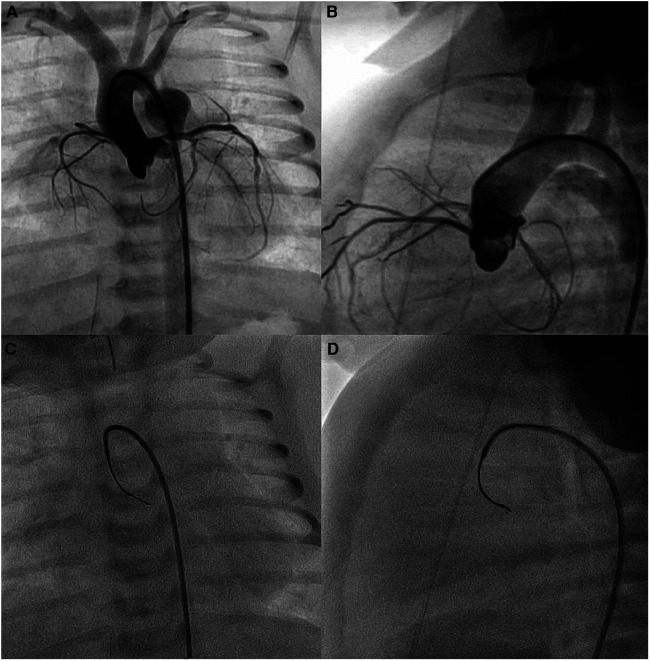
Baseline aortic root angiogram (**A,B**). SwiftNINJA is articulated perpendicular to the plane of the aortic valve root (to the left and posteriorly) to orient the coronary wire in the right direction and cross easily the valve (**C,D**).

In three patients with severe aortic valve stenosis (no. 4, no. 6, and no. 8), the plane of the aortic valve root was more vertically oriented, with a large right cusp and short left cusp. The use of SwiftNINJA gave us the right angulation of the catheter tip to orient the coronary wire in the right direction and easily cross the valve (Figure 3). In patient no. 17 with Damus-Kaye-Stansel repair, we used the SwiftNINJA to cross an apical trabeculated ventricular septal defect, create an arterio-venous circuit and close the defect from the jugular vein (Supplementary Video S1).

**Figure 4:**
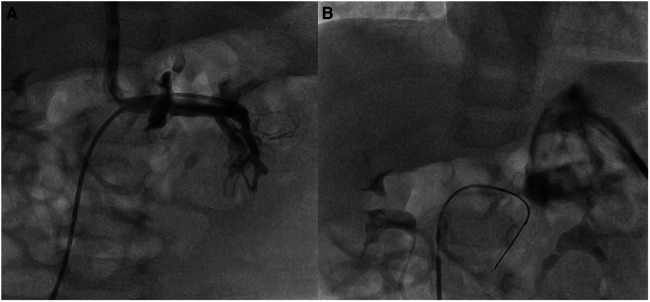
Patient no. 1 with type II abernathy malformation. The glide catheter is advanced deep inside the Abernathy vein acutely originating from the caval vein after being slid over the SwiftNINJA (**A**) SwiftNINJA is articulated at a 90-degree angle from the common trunk to engage deep inside the portal vein (**B**).

In two patients (no. 1 and no. 10) with Type II Abernathy malformations, the use of SwiftNINJA allowed us to access the acutely originating Abernathy vein from the caval vein, and also go deeper inside the liver to cannulate the best branch and rest the wire before advancing the glide over it (Figure 4). The procedure aimed to assess the patency of the portal circulation and the possibility of blocking partially the Abernathy vein to enhance the portal vein growth.

In two patients (no. 18 and no. 19) with pulmonary veins disease and small atrial defects, SwiftNINJA was very helpful in engaging specifically the difficult angle of the right lower pulmonary veins and dilating them. In patient no. 5 with a reconstructed pulmonary artery trunk and unifocalized collaterals, the distal branches were extremely narrowed and the SwiftNINJA was of great help in navigating the pulmonary tree safely and efficiently into the distal section. In patient no. 9, SwiftNINJA was very helpful in accessing transarterially the Blalock-Taussig shunt with an origin arising at an area close to a 90° curve of the aorta. In patient no. 13, the previously implanted stent in the right pulmonary artery was jailing completely the right lower lobe branch. The SwiftNINJA was used to anchor the catheter lumen in between the struts posteriorly and inferiorly and orient the wire in the right lower lobe branch for subsequent ballooning (Supplementary Video S2).

**Figure 5:**
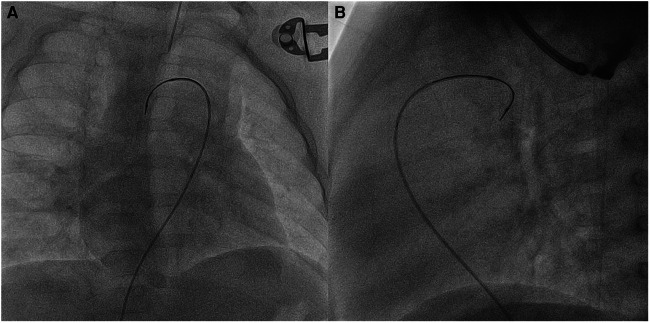
Patient no. 2. Right anterior oblique (**A**) and lateral (**B**) views showing SwiftNINJA angulated at a 90-degree angle to cross the ductal stent transvenously after crossing the ventricular septal defect and the ascending aorta.

In three patients (no. 2, no. 7, and no. 16) with previously stented arterial ducts, SwiftNINJA was applied to cross the ductal stents transvenously by going across the ventricular septal defect and through the ascending aorta (Figure 5 and Supplementary Video S3). We used the venous approach in these small patients because the French size is more flexible, in case a bigger balloon was needed for the dilation of the stent. In patient no. 11, the ductal stent was oriented upward and protruding in the descending aorta. We used the SwiftNINJA to engage the lumen of the stent without crossing the struts (Supplementary Video S4). In patient no. 14, we accessed transarterially the ductal stent and dilated the narrowed origin of the right pulmonary artery. SwiftNINJA was very helpful in re-accessing the left pulmonary artery, without crossing the stent struts and opening the struts to the left pulmonary artery.

The median applied tip angulation was 90 degrees (IQR, 85–95). No poor tip movement was identified. The device was found to be intuitive to use and a steep learning curve was not encountered. Adequate steerability was achieved even when selecting small and tortuous vessels and the device tracked appropriately when used with a guidewire. All procedures were subsequently completed. The overall median procedural time was 120 min (IQR, 112–138) and the median fluoroscopy time was 44 min (IQR, 27–71).

### 3.3. Immediate and short-term follow-up

There were no adverse events associated with the use of the SM. There was no general or vascular access complication. No device-related complications were recorded on the short-term follow-up.

## 4. Discussion

As the number of minimally invasive catheter procedures increases yearly, the need for advanced technology increases as well. The use of traditionally available catheterization hardware has been designed mainly for adults. However, experienced cardiac interventionists understand that adult and pediatric interventions can necessitate different management, affecting patient care and outcomes. Currently, there are few manufactured hardware specifically tailored for transcatheter use in newborns and infants with CHDs, pressuring interventional pediatric cardiologists to adapt commonplace and readily available devices for the minimally-invasive management of children. The recent introduction of SMs in the field of interventional radiology innovated procedural protocols (1, 2, 5–12). Until this time, this device has never been applied during transcatheter procedures in children with CHDs.

Several situations can create challenges to selective catheterization, such as acute angle branching, vessel tortuosity, small vessel diameter, severe vessel or valvular stenosis, poor trackability of the catheter and wire, and reduced operator skills. When dealing with challenges, one might have difficulty in selective catheterization or be forced to abandon the catheterization even if the shape of the guidewire and catheter is chosen to fit into the desired side branch. Even if a guidewire enters into the targeted branch, the microcatheter in some cases cannot follow the wire at the orifice or a guidewire may be forced out by the dynamics of the pushing manipulation. It is not uncommon to use multiple types of wires and catheters in a single case. Frequent hardware exchanges make it possible to use one wire/catheter combination for a certain task and switch to a different combination for a subsequent task while maintaining the same access. However, the major drawback of frequent material exchanges is the increased procedural time.

Increased procedural time has been associated with increased morbidity (14). This is especially relevant in small or critically ill patients. Catheter time is linked to procedural time and carries its risks. The longer a vessel is catheterized, the more prone it is to thrombosis or spasm. Increased intravascular manipulations also increase the risk of vascular dissection (15). These principles are more significant for small-caliber vessels. Additionally, increased procedural time is associated with increased radiation exposure (16). The cumulative effects of operator exposure are important to recognize and mitigate whenever possible.

Various devices and techniques have been developed to overcome the challenges of selective catheterization. Microcatheters with a pre-shaped tip, shaping of the tip with hot steam, creating a side hole or a cleft at the tip of the guiding catheter, and temporary balloon occlusion technique can facilitate selective catheterization. However, each technique has its pitfalls (17–19). More recently, SMs have been reported as efficient devices that allow operators to perform a broad spectrum of selective catheterization procedures in less time and radiation and with less contrast material exposure to the patient (5–7). SwiftNINJA is operator-guided and can be easily directed during catheterization without removing the catheter to reshape the tip. The use of SMs without or with a guidewire has been associated with a reduced number of hardware exchanges and decreased time to target vessels when compared to conventional microcatheter use (6). This would be expected to be associated with a decrease in complications such as guidewire-induced vascular spasms, arterial dissections, and even valvular tears. With all these advantages, the use of SMs has been rapidly expanded by some operators to challenging extravascular interventions (9–12, 20, 21).

### 4.1. Application in CHD interventions

In this study, we showed that SwiftNINJA SM was applied successfully in various anatomical scenarios for efficient super-selective catheterization and subsequent interventional procedures in children with CHDs. The inherent ability of SM to access smaller vascular lumen makes these devices an interesting choice when performing pediatric interventions. Articulating the tip to the same angle as the aortic valve plane helped us to advance the guidewire and the SwiftNINJA through the stenosis, avoiding excessive wire manipulations and the associated valvular trauma. This feature was also helpful in crossing smoothly an apical trabeculated ventricular septal defect and in navigating unusual tortuous intra-cardiac routes to access the arterial ducts from the venous side, thereby avoiding larger arterial introducers. The 3D steering function facilitates maneuvers across endovascular stents, to accurately cannulate the lumen without engaging the struts or to catheterize challenging jailed branches by crossing the correct struts (22). During bilateral implantation of pulmonary flow restrictors, the anatomy and angulation of the pulmonary bifurcation can be tricky sometimes. We don't want to take any risks after implanting the left pulmonary flow restrictor to have the wire going by mistake to the left pulmonary artery which can dislodge or affect the configuration of the freshly implanted left pulmonary flow restrictor, especially when the mouth of the device is a bit sticking out (13). Therefore, we think that SwiftNINJA SM can be used upfront to anticipate increased procedural time, contrast material, and radiation exposures concerning tricky vascular anatomies.

### 4.2. Extended possibilities

Placing catheters over wires has been the foundation of cardiac catheterization since the advent of the specialty. SMs provide the ability to perform complex procedures without the need for a wire, and in certain situations, without the need for a base catheter. This new concept may shift this paradigm and might be used to develop new catheterization techniques. The concept can be applied during transcatheter ductal closure in premature infants to navigate the right heart without damaging the tricuspid valve (23). SMs allow the operator to navigate tortuous vasculature via intermittent contrast injections while adjusting simultaneously the tip in the desired direction and advancing the catheter as necessary. By eliminating numerous wire exchanges and using “real-time road mapping” software, procedure time, radiation, and contrast doses, can be reduced (24). For example, this concept would potentially ease the distal navigation and subsequent treatment of coronary fistulas, abnormal arterial collaterals, or venous fistulas (25). During interstage catheterization of patients with modified surgical Norwood procedures, SM would also fasten transarterial cannulation of banded pulmonary arteries that originate usually high and posteriorly within the pulmonary homograft (26). It could also be a game changer for transvenous retrograde thoracic duct cannulation and embolization in Fontan patients with recurrent plastic bronchitis (27). In addition, Soyama et al. described a new technique of compact coil packing for the target vessels with the use of intentional folding by the bendable tip of the SM (8). This technique has been applied to effectively isolate a pseudoaneurysm with fewer coils, thus avoiding coil deviations and non-target embolization (28).

It has been also advocated that SMs should be considered when selective catheterization could not be completed despite using high-performance micro-guidewires. However, the mechanical support for deep insertion of the guidewire with an engaged microcatheter can be helpful in some anatomies. Due to increased market availability, its indications may shift, making it a primary procedural microcatheter rather than a problem-solving device. This also promotes the concept of the SM as a prospectively invaluable device to utilize, more especially when procedure time dictates the outcome of patient prognosis (7).

### 4.3. Limitations

Even though SwiftNINJA is an epoch-making device, this SM has two main technical limitations. First, the tip of the catheter is 2.4 Fr and might not be useful in tiny branches. Second, the tip movement is limited within the two-dimensional plane. The torquability of this catheter can aim the tip in a three-dimensional plane, yet it is not as stiff as the standard wires. In tortuous vessels, the friction between the catheter and the vessel wall might impede the transmission of rotational forces, and supportive use of the guidewire might be necessary.

With the pressure to reduce healthcare costs, some might say that the MC is too expensive for routine use in pediatric catheterization laboratories. What should be considered is the time that using a device to make the procedure faster and more efficient could save. The time spent on any given case can have an exponential financial impact due to staffing, time away from other procedures, and supplies used. This can impact radiation and contrast material safety together with the workflow perspective. In our series, SMs were applied as a bailout option in most cases after extensive attempts with various pre-shaped catheters, microcatheters, and wires failed. However, the increased throughput allowed by shorter procedure times and cost savings from using fewer microcatheters and guidewires may offset the higher unit cost of SMs. Analysis of the cost-effectiveness of SMs is a topic for future research.

## 5. Conclusions

This is the first reported intravascular use for the SwiftNINJA SM in cardiac catheterization procedures of children with congenital heart lesions. Our findings suggest that SwiftNINJA SM are efficient devices with clinical benefits and a positive impact on procedural success and radiation exposure.

## Data Availability

The raw data supporting the conclusions of this article will be made available by the authors, upon request, to any qualified researcher.

## Supplementary material

Supplementary Video 1 Patient no. 17 with a Damus-Kaye-Stansel repair. SwiftNINJA is used to cross an apical ventricular septal defect and create an arteriovenous circuit for subsequent defect closure from the jugular vein.

Supplementary Video 2 Patient no. 13. Right lower lobe branch was jailed completely by the previously implanted stent in the right pulmonary artery The SwiftNINJA was used to anchor the catheter lumen in between the struts posteriorly and inferiorly and orient the wire in the right lower lobe branch for subsequent ballooning.

Supplementary Video 3 Patient no. 16. SwiftNINJA applied to cross the ductal stent transvenously.

Supplementary Video 4 Patient no. 11. Ductal stent oriented vertically upward and protruding slightly in the descending aorta. SwiftNINJA articulated at a 140-degree angle to engage the lumen of the stent without crossing the struts.

## References

[1] Martin JT Hulsberg PC Soule E Shabandi M Matteo J. Welcome to the new era: a completely wireless interventional procedure. Cureus. (2018) 10(9):e3337. 10.7759/cureus.3337 30473970 PMC6248806

[2] Dudeck O. Safety and efficacy of target vessel catheterization with the new steerable microcatheter direxion compared with a standard microcatheter: a prospective, preclinical trial. Cardiovasc Intervent Radiol. (2014) 37(4):1041–6. 10.1007/s00270-014-0918-x 24849303

[3] Wang L Yuan S Borgquist R Höijer CJ Brandt J. Coronary sinus cannulation with a steerable catheter during biventricular device implantation. Scand Cardiovasc J. (2014) 48(1):41–6. 10.3109/14017431.2013.875623 24432887

[4] Er F Yüksel D Hellmich M Gassanov N. Comparison of conventional versus steerable-catheter guided coronary Sinus lead positioning in patients undergoing cardiac resynchronization device implantation. PLoS One. (2015) 10(11):e0143292. 10.1371/journal.pone.0143292 26599637 PMC4658090

[5] Inaba Y Arai Y Sone M Aramaki T Osuga K Tanaka H Experiments for the development of a steerable microcatheter. Cardiovasc Intervent Radiol. (2017) 40(12):1921–6. 10.1007/s00270-017-1789-8 28879604

[6] Hoffmann JC Minkin J Primiano N Yun J Eweka A. Use of a steerable microcatheter during superselective angiography: impact on radiation exposure and procedural efficiency. CVIR Endovasc. (2019) 2(1):35. 10.1186/s42155-019-0078-9 32026024 PMC6966367

[7] Harmon TS Hulsberg PC McFarland JR 3rd Villescas VV Matteo J. Time is brain: the future for acute ischemic stroke management is the utilization of steerable microcatheters for reperfusion. Cureus. (2019) 11(1):e3842. 10.7759/cureus.3842 30891383 PMC6411324

[8] Soyama T Yoshida D Sakuhara Y Morita R Abo D Kudo K. The steerable microcatheter: a new device for selective catheterisation. Cardiovasc Intervent Radiol. (2017) 40(6):947–52. 10.1007/s00270-017-1579-3 28138724

[9] Eadie E Harmon TS Soule E Hulsberg PC Shabandi M Matteo J. A novel nonvascular application of the steerable microcatheter. Cureus. (2018) 10(10):e3469. 10.7759/cureus.3469 30585285 PMC6300387

[10] Padilla RM Hulsberg PC Soule E Harmon TS Eadie E Hood P Against the odds: a novel technique to perform cholangiography from a percutaneous approach through the cystic duct. Cureus. (2018) 10(11):e3577. 10.7759/cureus.3577 30656081 PMC6333255

[11] Harmon TS Kee-Sampson J Hester TS Bashir S Matteo J. Pediatric traumas and paradigm shifts: the necessary adaption of the steerable microcatheter in pediatric interventional radiology. Cureus. (2019) 11(2):e4125. 10.7759/cureus.4125 31049274 PMC6483116

[12] Kassimis G Kontogiannis N Raina T. Steerable microcatheters for complex percutaneous coronary interventions in octogenarians: from venture to swift ninja. J Geriatr Cardiol. (2019) 16(1):54–9. 10.11909/j.issn.1671-5411.2019.01.004 30800152 PMC6379244

[13] Haddad RN Bentham J Adel Hassan A Al Soufi M Jaber O El Rassi I Outcomes of manually modified microvascular plugs to pulmonary flow restrictors in various congenital heart lesions. Front Cardiovasc Med. (2023) 10:1150579. 10.3389/fcvm.2023.1150579 37492157 PMC10363685

[14] Cheng H Clymer JW Po-Han Chen B Sadeghirad B Ferko NC Cameron CG Prolonged operative duration is associated with complications: a systematic review and meta-analysis. J Surg Res. (2018) 229:134–44. 10.1016/j.jss.2018.03.022 29936980

[15] Mayer J Tacher V Novelli L Djabbari M You K Chiaradia M Post-procedure bleeding in interventional radiology. Diagn Interv Imaging. (2015) 96(7-8):833–40. 10.1016/j.diii.2015.06.009 26138359

[16] Haddad RN Rizk C Saliba Z Farah J. Percutaneous closure of ventricular septal defects in children: key parameters affecting patient radiation exposure. Am J Cardiovasc Dis. (2021) 11(1):65–72. 33815921 PMC8012278

[17] Kiyosue H Hori Y Matsumoto S Okahara M Tanoue S Sagara Y Shapability, memory, and luminal changes in microcatheters after steam shaping: a comparison of 11 different microcatheters. AJNR Am J Neuroradiol. (2005) 26(10):2610–6. 16286410 PMC7976220

[18] Miyayama S Matsui O Akakura Y Yamamoto T Fujinaga Y Koda W Use of a catheter with a large side hole for selective catheterization of the inferior phrenic artery. J Vasc Interv Radiol. (2001) 12(4):497–9. 10.1016/s1051-0443(07)61890-9 11287538

[19] Abdelsalam ME Mahvash A Avritscher R McRae SE Odisio BC. Balloon-assisted flow diversion and selective catheterization of target vessels for hepatic transarterial embolization. J Vasc Interv Radiol. (2016) 27(2):283–5. 10.1016/j.jvir.2015.10.022 26830942

[20] Zhang D Zheng L Rao Q Zhao Z. The SwiftNINJA steerable microcatheter for continuous hepatic artery infusion chemotherapy for the treatment of advanced intrahepatic cholangiocarcinoma. Asian J Surg. (2022) 45(1):656–8. 10.1016/j.asjsur.2021.11.002 34810117

[21] Hamaguchi S Michigami Y Inoue M Tsukamoto K Wada S Ogawa Y. Successful treatment of chylous ascites by superselective embolization of the inflowing lymphatic vessels using a steerable microcatheter: a case study. Radiol Case Rep. (2022) 17(9):3205–8. 10.1016/j.radcr.2022.06.011 35801127 PMC9253846

[22] Manica JL Piazza L Butera G. The use of a wire control catheter to treat complex pulmonary artery or vein anatomy. J Invasive Cardiol. (2012) 24(7):E148–52. 22781486

[23] Meot M Gaudin R Szezepanski I Bajolle F Bonnet D Malekzadeh-Milani S. Transcatheter patent arterial duct closure in premature infants: a new technique to ease access to the patent arterial duct, with particular benefit for the tricuspid valve. Arch Cardiovasc Dis. (2021) 114(6-7):482–9. 10.1016/j.acvd.2021.06.002 34312100

[24] Glöckler M Halbfaβ J Koch A Achenbach S Dittrich S. Multimodality 3D-roadmap for cardiovascular interventions in congenital heart disease–a single-center, retrospective analysis of 78 cases. Catheter Cardiovasc Interv. (2013) 82(3):436–42. 10.1002/ccd.24646 22936634

[25] Haddad RN Bonnet D Malekzadeh-Milani S. Embolization of vascular abnormalities in children with congenital heart diseases using medtronic micro vascular plugs. Heart Vessels. (2022) 37(7):1271–82. 10.1007/s00380-021-02007-6 35088203

[26] Pontailler M Gaudin R Lenoir M Haydar A Kraiche D Bonnet D Hypoplastic left heart syndrome: a novel surgical strategy for small-volume centres? Eur J Cardiothorac Surg. (2017) 51(5):1003–8. 10.1093/ejcts/ezx021 28329111

[27] Haddad RN Dautry R Bonnet D Malekzadeh-Milani S. Transvenous retrograde thoracic duct embolization for effective treatment of recurrent plastic bronchitis after fontan palliation. Catheter Cardiovasc Interv. (2023) 101(5):863–9. 10.1002/ccd.30611 36861752

[28] Umakoshi N Arai Y Sone M Sugawara S. Compact coil packing using a steerable microcatheter for a giant wide-necked pulmonary artery pseudoaneurysm. Interact Cardiovasc Thorac Surg. (2019) 28(5):826–7. 10.1093/icvts/ivy332 30561622

